# Social deprivation among socio-economic contrasted french areas: Using item response theory analysis to assess differential item functioning of the EPICES questionnaire in stroke patients

**DOI:** 10.1371/journal.pone.0230661

**Published:** 2020-04-02

**Authors:** Adrien Guilloteau, Christine Binquet, Abderrahmane Bourredjem, Isabelle Fournel, Marie Laure Lalanne-Mistrih, Mathieu Nacher, Devi Rochemont, André Cabie, Emmanuelle Mimeau, Caroline Mislin-Tritsch, Julien Joux, Annie Lannuzel, Claire Bonithon-Kopp, Yannick Béjot, Hervé Devilliers, the INDIA study group

**Affiliations:** 1 Clinical Epidemiology Unit, INSERM, CIC 1432, University Hospital of Dijon, Epidemiology and infection control unit, Bourgogne-Franche-Comté University, Dijon, France; 2 INSERM, CIC 1424, Clinical Epidemiology Unit – Pointe-à-Pitre, University Hospital of Pointe-à-Pitre – Pointe-à-Pitre, University of West Indies – Pointe-à-Pitre, Guadeloupe, France; 3 INSERM, CIC 1424, Clinical Epidemiology Unit, Hospital Andrée Rosemon, CIC 1424, Clinical Epidemiology Unit, University of French Guiana – Cayenne, Guyane, France; 4 INSERM, CIC 1424, Clinical Epidemiology Unit – Fort-de-France, University of West Indies, EA4537 – Fort-de-France, University Hospital of Martinique – Fort-de-France, Martinique, France; 5 Hospital Andrée Rosemon – Cayenne, Guyane, France; 6 Western Guyana Hospital – Saint Laurent du Maroni, Guyane, France; 7 University Hospital of Martinique – Fort-de-France, Martinique, France; 8 INSERM, CIC 1424, Clinical Epidemiology Unit – Pointe-à-Pitre, University Hospital of Pointe-à-Pitre – Pointe-à-Pitre, University of West Indies – Pointe-à-Pitre, Institute for Brain and Spinal Cord Disorders, ICM, UMR 1127, Paris, France; 9 Neurology Department and Dijon Stroke Registry, University Hospital of Dijon, Dijon, France; Catholic University of Korea College of Medicine, REPUBLIC OF KOREA

## Abstract

**Background:**

Multiple approaches have been proposed to measure low socio-economic status. In France the concept of precariousness, akin to social deprivation, was developed and is widely used. EPICES is a short questionnaire that was developed to measure this concept. This study aimed to evaluate Differential Item Functioning (DIF) in the EPICES questionnaire between contrasted areas: mainland France, French West Indies (FWI) and French Guiana (FG).

**Methods:**

The population was taken from the INDIA study, which aimed to evaluate the impact of social inequalities on stroke characteristics and prognosis. Eligible people were patients referred to neurology or emergency departments for a suspicion of stroke. We assessed the DIF using hybrid ordinal logistic regression method, derived from item response theory.

**Results:**

We analysed 1 553 stroke patients, including 768 from FWI (49.5%), 289 from FG (18.6%) and 496 from mainland (31.9%). We identified five items with a moderate to large DIF in area comparisons: “meeting with a social worker”, “complementary health insurance”, “home-owning”, “financial difficulties” and “sport activities”. Correlation between EPICES score and the latent variable was strong (r = 0.84).

**Conclusion:**

This is the first attempt to assess the DIF of the EPICES score between different French populations. We found several items with DIF, which can be explained by individual interpretation or local context. However, the DIFs did not lead to a large difference between the latent variable and the EPICES score, which indicates that it can be used to assess precariousness and social deprivation between contrasted areas.

## Introduction

Since the Second World War, socio-economic inequalities have shown a stable or rising trend between the wealthiest and the poorest populations, even in countries with a large welfare budget [1]. For example, in France, a well-known welfare state, life expectation differences between manual workers and managerial and professional jobs is still 6 years for men and 3 years for women, and are at the same level as that in the eighties [2]. Recent research has focused on the relationship between low socio-economic status and the occurrence or poor prognosis of many diseases. An unhealthy lifestyle, lack of knowledge about disease and limited access to the health care system in the most disadvantaged have been mentioned to explain this relationship [3–5]. These results demonstrate that taking into account the effect of social status or poverty is a truly critical issue in epidemiological studies [6].

Poverty is often measured through income or financial resources. However, the strong cultural taboo on revealing earnings in some countries is a caveat to the use of this approach in epidemiological studies [7]. Moreover, this approach fails to account for the multidimensional aspects of poverty and particularly its social dimension.

In the late seventies, Townsend worked on low socio-economic status through a new approach, relative deprivation, which he defined as a lack of resources to sustain the lifestyle that is common or approved by society [8]. Relative deprivation is considered relative in that it is defined relatively to other people in the same society; this can be opposed to an absolute approach of deprivation or poverty, which consists in defining an income threshold based on individual needs. Later, Townsend made a distinction between material deprivation (lack of resources, commodities) and social deprivation (lack of interaction with the rest of the society) [9]. Since its development, the concept of relative deprivation and its definition have been widely adopted in clinical research.

Another approach to low socio-economic status was initiated in France: the concept of precariousness (translated from the French word “précarité”). It was developed to identify people close to falling into poverty. People in a precarious socio-economic situation still participate in society, and could, with the right action, be saved from falling into poverty. By targeting such populations, social interventions are easier to implement (this population is easier to target and the those concerned are more willing to accept preventive measures than is the case with more severely deprived people) with more achievable aims (prevent the fall instead of actively recovering from it). The most popular definition for precariousness was elaborated in 1987 by Wresinsky [10]. He defined it as an unstable state concerning one or more basic securities (a job, health, family status…) that prevented people from enjoying fundamental rights and that could lead to poverty. As it is more encompassing, precariousness is more common than poverty: it was reported to be as high as 25% for France [3,11]. This concept is rooted in the French context: people in precarious situations are often avoiding poverty thanks to welfare benefits. Contrary to social deprivation, precariousness is defined in an absolute sense but they share close ties.

A short questionnaire was devised to rapidly assess the level of precariousness at the individual level, in a large sample of the French population [5,12,13]: the EPICES score (“Évaluation de la Précarité et des Inégalités de santé dans les Centres d’Examen de Santé”), published in 2006. The aim of EPICES was to detect the level of precariousness in people consulting in health centres, but it was also used in epidemiological studies [14–17]. This score was developed using classical test theory and validated against other measurements in a heterogeneous population [18,19]. As such it can be used in various cultural contexts. However, differential item functioning (DIF) in EPICES was never assessed. DIF corresponds to a differential item interpretation or difficulty between groups. Differences in home-ownership between rural and urban people is a classic example in the literature on deprivation [20,21]: as houses in the countryside are cheaper than houses in urban areas, for the same level of poverty, rural people will more frequently be home owners than is the case for city dwellers. This can lead to a difference in deprivation scores, which is not due to a difference in the level of deprivation. Thus, if a DIF is present for a covariate (age, area of residence, gender) in a questionnaire, comparisons between results for each category of this covariate are invalid. We took the opportunity of the INDIA study on the prognosis following stroke in metropolitan France, the French West Indies (FWI) and French Guiana (FG) [17], to evaluate the DIF of each item of the EPICES questionnaire according to geographical areas, gender and age categories.

## Methods

### Study patients

The INDIA (“INégalités sociales et pronostic des accidents vasculaires cérébraux à DIjon et en Antilles-Guyane”) study is a French multicentre prospective cohort which aims to evaluate the impact of social inequalities on stroke characteristics and prognosis [17]. Patients were recruited between June 2011 and October 2014, from six centres located in three geographical areas chosen for their socioeconomic disparities (Dijon, Burgundy; Fort-de-France and Pointe-à-Pitre, FWI; Cayenne, Saint Laurent du Maroni and Kourou, FG), including three neurology departments of university hospital and three emergency department. Briefly, eligible people were patients older than 17 years admitted for an acute stroke diagnosed according the World Health Organization definition. Further inclusion criteria were: stroke confirmed by imaging and patients able to be interviewed (personally or via support persons). Patients who had a history of symptomatic stroke, a short-term life-threatening condition, or whom we were unable to contact during follow-up (12 months) were excluded. At the end of the recruitment period, 1 573 patients were included in the cohort. Oral or written consent was not required by French legislation at the initiation of the study. The protocol was approved by the Burgundy Ethics Committee (CPP Est 1, 16 May 2010) without requiring consent; all patients received an information sheet about the study. The study did not include minors.

### Collected data

Multiple socio-economic data were collected during the INDIA study: ethnic origin (geographic origin of parents or ancestors), marital status, employment status, socioprofessional status, highest diploma obtained, home-ownership, payment of income tax, health insurance, receipt of social assistance benefits and the EPICES score.

The EPICES questionnaire was administered to all patients as soon as possible after the stroke. This questionnaire was designed to assess the level of precariousness by the means of 11 binary items (Table 1). Among these 11 items, two are very frequent in material deprivation scales: items #4–5. Six are related to social deprivation: items #1, #3, #6–9. One is related to health and financial difficulties: item #1. The last two [#10–11] are specific to precariousness scales. The final score can vary from 0 (no precariousness), to 100 (extreme precariousness), each item having its own weight. The authors suggested two cut-offs to categorize people as in a precarious situation, according to the last quintiles in the validation sample: 30.17 and 48.5.

**Table 1:** EPICES items and proportion of yes answers by subpopulations (INDIA study, N = 1 553).

EPICES items	% of “yes” by subpopulation							
	Dijon	French Guiana	French West Indies	Men	Women	< 65 years old	≥ 65 years old	Whole sample
Group size	496	289	768	890	663	891	662	1553
#1. Do you sometimes meet a social worker?	12,9%	12,1%	9,1%	**8,4%**	**14,2%**	10,4%	11,5%	10,9%
#2. Do you have complementary health insurance?	**94,2%**	**62,3%**	**74,9%**	**74,2%**	**84,8%**	**81,1%**	**75,4%**	78,7%
#3. Do you live as a couple?	**65,5%**	**51,6%**	**49,0%**	**65,5%**	**40,3%**	53,3%	56,6%	54,7%
#4. Are you a homeowner?	**65,9%**	**41,9%**	**67,2%**	61,7%	62,6%	**72,6%**	**47,9%**	62,1%
#5. Are there periods in the month when you have real financial difficulties to meet your needs (food, rent, electricity)?	**20,0%**	**57,8%**	**33,1%**	34,5%	32,1%	**25,9%**	**43,7%**	33,5%
#6. Have you done any sports activities in the last 12 months?	**19,6%**	**29,8%**	**36,3%**	**33,1%**	**25,2%**	**22,6%**	**39,4%**	29,7%
#7. Have you been to any shows over the last 12 months?	**41,1%**	**22,8%**	**28,3%**	30,9%	32,0%	**23,1%**	**42,4%**	31,4%
#8. Have you been on holiday over the last 12 months?	**40,5%**	**28,7%**	**31,0%**	34,0%	33,0%	**26,6%**	**43,1%**	33,6%
#9. Have you seen any family member over the last six months (other than your parents or children)?	84,9%	84,8%	84,0%	82,9%	86,4%	83,4%	85,8%	84,4%
#10. If you have difficulties, is there anyone around you who could take you in for a few days?	86,9%	81,3%	82,4%	83,3%	84,2%	83,5%	83,8%	83,6%
#11. If you have difficulties, is there anyone around you who could provide you with material assistance?	82,5%	79,6%	82,9%	80,6%	84,3%	82,8%	81,3%	82,2%

*Significant differences at alpha 0*.*05 determined by a Chi*^*2*^ *test are showed in bold*.

Clinical data were also recorded, including known and suspected stroke risk factors, characteristics of the stroke, clinical state before the stroke and after the stroke.

### Statistical analysis

We first described answers for items in percentages for the whole population and for each subpopulation. For age, two categories were considered: less than 65 years old; aged 65 and over. Then we conducted the DIF analysis according to item response theory (IRT). Briefly, IRT analysis makes it possible to estimate the patient’s “ability” also called the latent variable (i.e. the level of precariousness), and the item “difficulty” (i.e. the level of precariousness at which a patient has 50% probability of “yes” answers) on the same logit scale. In this study, the analyses were conducted using a Graded Response Model (GRM), because of its ability to estimate the discriminant capacity of an item, represented by the slope: for two items with the same difficulty, people with a low ability will have a higher probability of answering yes to the item with the lower slope (i.e. the lower discriminant capacity) and people with a high ability will have a lower probability of answering yes to the same item.

IRT conditions were tested. Item fit was assessed using the infit and outfit statistics for each item. Acceptable ranges were 0.7 to 1.3 [22]. Principal Component Analysis (PCA) of the residual and residual Spearman correlation matrix was examined to ensure unidimensionality and the local independence of items. If necessary, we planned to exclude items resulting in a bad fit or local dependence to be able to pursue the analysis.

Responses to EPICES items 1 and 5 had to be reversed for the purpose of the analysis (“no” corresponding to a higher precariousness level for other items). This was done after descriptive analysis, thus Table 1 is not affected by this change, but all of the other analyses are.

To evaluate differential item functioning we used a hybrid ordinal logistic regression (HOLR) developed in the *lordif* package for R software by Choi et al [23], which relies on the GRM. This approach allowed us to differentiate between uniform and non-uniform DIF: uniform DIF corresponds to a difference in difficulty (the item is always more difficult in one population than in another) whereas non-uniform DIF is a difference in difficulty and slope (the difference in difficulty of the item between two populations can vary according to the latent variable level).

For each item, three different models were compared: the first model with no DIF, the second model with a uniform DIF, and the third model with a non-uniform DIF. The presence of DIF was assessed by using a log likelihood ratio test (alpha level at 0.01). Then differences in Mc Fadden R^2^ (or ΔR^2^) were used to determine DIF magnitude and type (uniform or non-uniform). Differences in R^2^ under 0.035 were deemed negligible, between 0.035 and 0.07 moderate, and above 0.07 important [22].

To assess classification bias for the EPICES score, we calculated the individual level of the latent variable, hypothesized to be precariousness, according to parameters obtained in the IRT analysis (with and without taking into account DIF) and compared it with the total EPICES score, using Pearson correlations. As the level of the latent variable represents the true value of precariousness, it allows us to evaluate if the score obtained in EPICES is close to the true value and if DIFs change significantly this relationship. We also compared the level of the latent variable estimated with and without DIF to assess the impact of the DIF on the latent variable estimation.

We also assessed internal consistency using Cronbach’s alpha [24] and the underlying structure with a multiple correspondence analysis (MCA) on answers, to analyse sub-dimensions of the questionnaire. These analyses were done on the whole sample and on geographical area groups. The number of dimensions to retain was determined using a scree plot, cumulative percentages of explained variance and interpretability. To be able to correctly interpret results, we applied a varimax-type rotation for MCA developed by Chavent et al [25]. All of the analyses were done using R v3.3.

All of the analyses were done using R v3.3.

## Results

### Population description

Among the 1 573 patients recruited in the INDIA study, 1 553 completed all items of the EPICES score. Detailed characteristics are presented in Table 2. The percentages of ‘yes’ answers are reported in Table 1. In our sample, 53.2% of patients had an EPICES score above 30, and 23.9% above 48.5.

**Table 2:** Population characteristics (INDIA study, N = 1 553).

		Median [Q1;Q3] or size (percentage)	Missing data
**socio-demographic characteristics**	Age at inclusion	68 [56;78]	0
	Age ≥ 65 years old	891 (57.4%)	0
	Men	890 (42.7%)	0
	Inclusion center		0
	- Dijon	496 (31.9%)	
	- Fort-de-France	400 (25.7%)	
	- Pointe-a-Pitre	368 (23.7%)	
	- French Guyana	289 (18.6%)	
	Origins		0
	- Parents of european origins only	534 (34.4%)	
	- Parents of sub-saharean origins only	495 (31.9%)	
	- Other	412 (26.5%)	
	- Unknown	112 (7.2%)	
	Married	853 (55.1%)	6
	EPICES score > 30	808 (52.0%)	0
**Vascular risk factors**	High blood pressure (before stroke)	571 (36.8%)	2
	Hypercholesterolemia	1203 (77.7%)	5
	Body Mass Index	26.0 [23.2;29.4]	217
	Diabete	1188 (76.5%)	1
	Current smoker	243 (15.7%)	8
	Alcohol consumption (≥2 glass per day)	208 (13.6%)	18
**Stroke characteristics**	NIHSS	6 [3;13]	21
	Type of stroke		0
	- Ischaemic stroke	1312(84.4%)	
	- Hemorrhagic stroke	228 (14.7%)	
	- Subarachnoid hemorrhage	13 (0.8%)	

*NIH*: *National Institutes of Health Stroke Scale*

### IRT conditions

Infit and outfit were between acceptable range values for all items. Unidimensionality was verified as there was no major component retained in the PCA analysis on the residuals (Eigen-value between 1.5 and 3 for the first component, according to the group, and decreasing slowly for the following). Local independence was assessed by Spearman correlations on residuals for the whole sample and for each group. Correlations between residuals were very high for items #10 and #11 (r = 0.601). Similar results were observed in the sub-group analysis (by geographic area, age and gender, S1 Data). The only exceptions were observed for the Dijon area, where two other strong correlations (between item #2 and #1 and between item #2 and #9) were highlighted (r > 0.4) despite being lower than for items #10 and #11.

As the IRT condition for local independence was not met due to the last item, #11, we chose to remove it from the DIF analysis. After reassessing conditions with only 10 items, local independence was improved (S4).

### DIF analysis: Geographical areas

**Fig 1:**
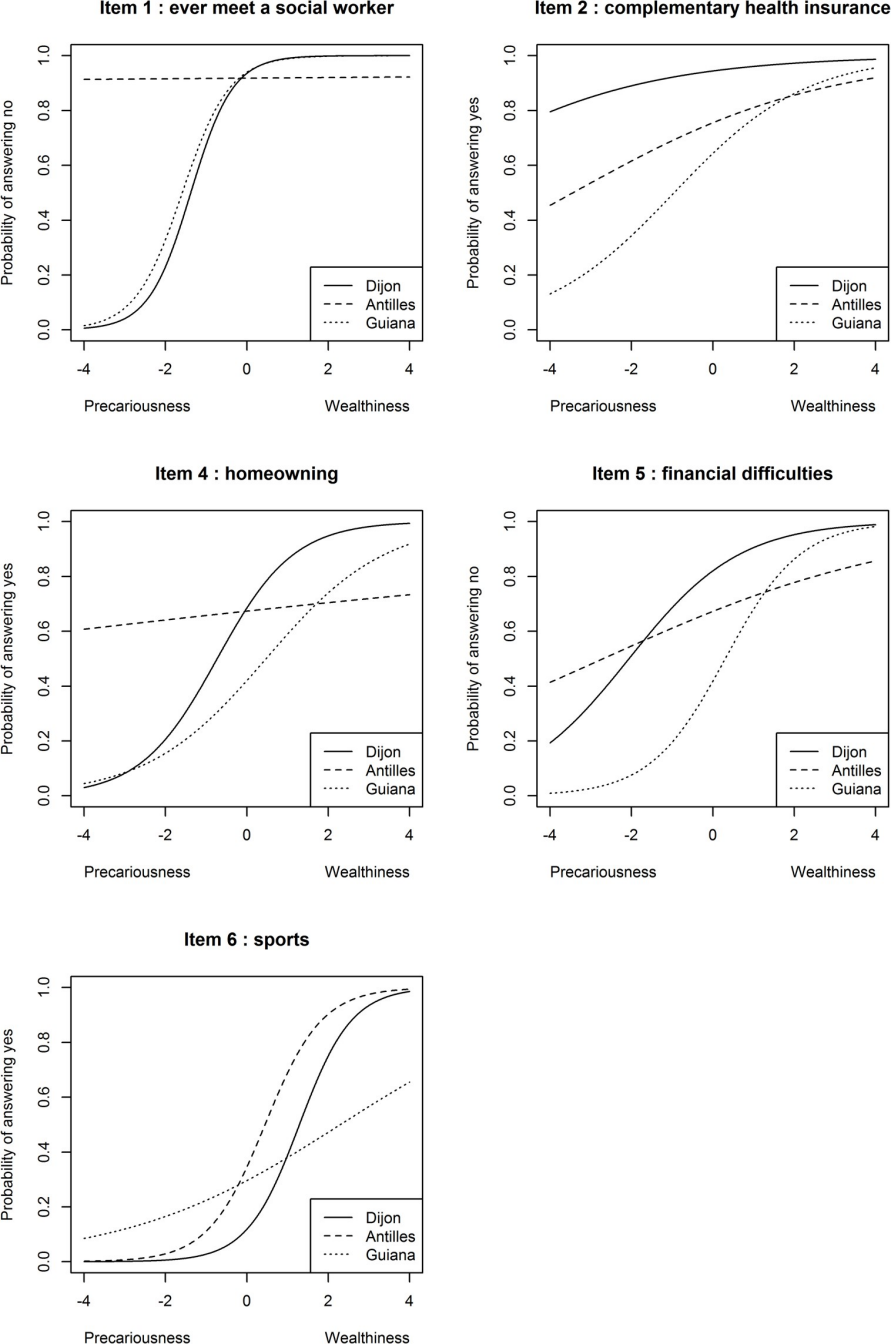
probability of answering according to level of precariousness for items with DIF in geographical areas analysis (INDIA study).

We assessed differential functioning items by comparing Dijon and FWI groups, Dijon and FG groups and FWI and FG groups. In these comparisons, we detected 10, 6 and 5 items, respectively, with a DIF (Table 3 and Fig 1). We found two items with a moderate DIF and two with an important DIF in the first comparison (respectively, items #4–6 and #1–2) and second comparison (respectively, items #2–5 and #4–6). In the third comparison, we found two items with a moderate DIF and one important (respectively, items #4–5 and #1).

**Table 3:** DIF significance and intensity for each comparison (INDIA study N = 1553).

Items	Labels	LRT test		DIF intensity	
		Χ_21_	Χ_32_	ΔR^2^_21_	ΔR^2^_32_
Dijon vs French West Indies					
**#1**	Social worker	0.016	<0.001	-	**0.116**
**#2**	Complementary health insurance	<0.001	0.916	**0.074**	-
**#3**	Couple	<0.001	<0.001	<0.035	<0.035
**#4**	Homeowner	0.448	<0.001	-	**0.045**
**#5**	Financial difficulties	<0.001	0.010	<0.035	-
**#6**	Sports activities	<0.001	0.124	**0.056**	-
**#7**	Shows	<0.001	<0.001	<0.035	<0.035
**#8**	Holidays	0.001	0.545	<0.035	-
**#9**	Meeting with family	0.943	<0.001	-	<0.035
**#10**	Relation that can give shelter	0.044	<0.001	-	<0.035
Dijon vs French Guiana					
**#1**	Social worker	0.283	0.278	-	-
**#2**	Complementary health insurance	<0.001	0.044	**0.172**	-
**#3**	Couple	0.001	0.015	<0.035	-
**#4**	Homeowner	<0.001	0.043	**0.038**	-
**#5**	Financial difficulties	<0.001	0.008	**0.124**	<0.035
**#6**	Sports activities	<0.001	<0.001	<0.035	**0.051**
**#7**	Shows	<0.001	0.586	<0.035	-
**#8**	Holidays	0.011	0.927	-	-
**#9**	Meeting with family	0.656	0.320	-	-
**#10**	Relation that can give shelter	0.090	0.179	-	-
French West Indies vs French Guiana					
**#1**	Social worker	0.240	<0.001	<0.035	**0.092**
**#2**	Complementary health insurance	<0.001	0.002	<0.035	<0.035
**#3**	Couple	0.201	0.073	-	-
**#4**	Homeowner	<0.001	<0.001	**0.037**	<0.035
**#5**	Financial difficulties	<0.001	<0.001	**0.035**	<0.035
**#6**	Sports activities	0.133	<0.001	-	<0.035
**#7**	Shows	0.217	0.013	-	-
**#8**	Holidays	0.838	0.599	-	-
**#9**	Meeting with family	0.217	0.122	-	-
**#10**	Relation that can give shelter	0.634	0.533	-	-

*Moderate (*Δ*R*^*2*^*>0*.*035 et ≤0*.*07) and important (ΔR*^*2*^*>0*.*070) DIF are shown in bold*.

*DIF intensity was not assessed when LRT test was not significant*.

*Index*_*21*_ *refers to the comparison between the model without DIF and the model with a uniform DIF*, *index*_*32*_ *to the comparison between the model with a uniform DIF and the model with a non-uniform DIF*

### DIF analysis: Gender and age

**Fig 2:**
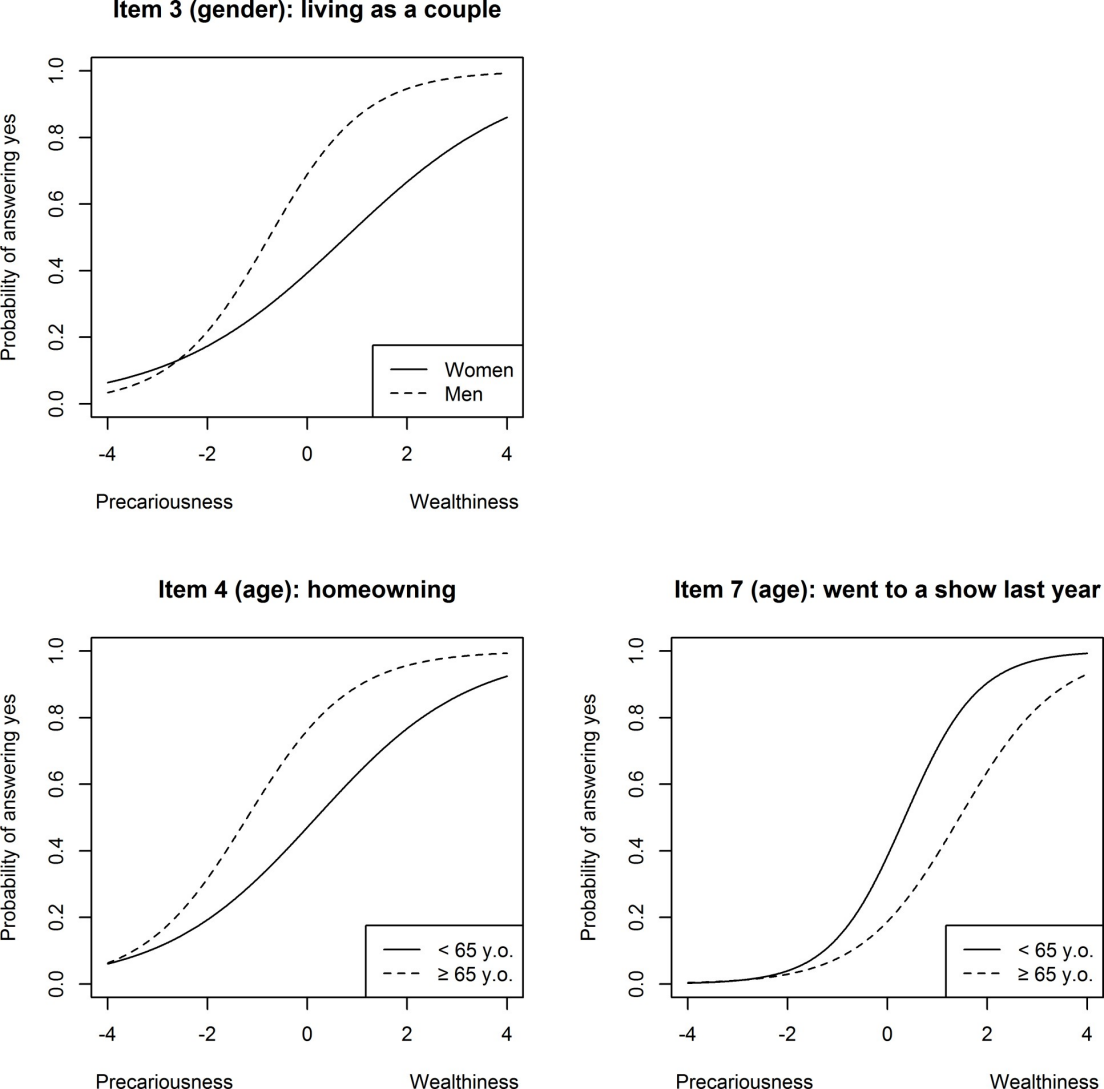
Probability of answering according to level of precariousness for items with DIF in age and gender analysis (INDIA study).

Five and six items had a DIF regarding gender and age, 4 were negligible in both analysis. In the analysis according to gender item 3 showed a moderate uniform DIF (Fig 2): living as a couple was associated with a lower difficulty in men (ΔR^2^ = 0.0606). We detected two DIF items in the analysis between age groups (item #4 and #7, both moderate and uniform): home-ownership (#4) was associated with a higher difficulty in the younger group (ΔR^2^ = 0.0628) whereas older people had a higher difficulty on item #7 (“shows”– ΔR^2^ = 0.0407).

### Reliability and MCA

Cronbach’s alpha was low (0.59 with 11 items, 0.54 with 10 items, in the whole sample). It was higher for the Dijon centre, and in patients below 65 years old (0.60 for both with 10 items), and lower for the FWI group (0.47 with 10 items).

Results of the MCA analysis are shown in Table 4. For each group, four dimensions were selected. After rotation, the results were consistent across groups, with or without the eleventh item. Overall, when considering the whole sample, four different dimensions could be considered: a first one, social withdrawal (SW), with a high contribution of the last two items (or only the tenth when the eleventh was not included) and a moderate contribution of the ninth; a second, leisure activities (LA) which included items #6 to #8 (with a higher contribution of item #7 and #8 in the FG and West Indies groups).

**Table 4:** % of variance and item contribution resulting from the MCAs after rotation, on the whole sample as well as in geographical areas groups, with 11 items and 10 items (INDIA study, N = 1553).

	**Whole sample**				**Dijon**				**French Guiana**				**French West Indies**			
	**SW**	**LA**	**HS**	**FD**	**SW**	**LA**	**HS**	**FD**	**SW**	**LA**	**Dim 3**	**Dim 4**	**SW**	**LA**	**Dim 3**	**Dim 4**
**% of variance**	17.9	14.2	12.2	11.5	16.9	15	14.4	10.2	17.7	13.1	12.9	11.6	19.4	14.6	11.4	10.7
**#1.**	-	-	0.592	-	-	-	0.479	-	-	-	-	0.501	-	-	-	0.661
**#2.**	-	-	-	0.668	-	-	-	0.583	-	-	0.392	-	-	-	0.567	-
**#3.**	-	-	0.219	-	-	-	0.507	-	-	-	0.347	-	-	-	0.405	-
**#4.**	-	-	0.379	-	-	-	0.461	-	-	-	0.29	-	-	-	-	0.264
**#5.**	-	-	-	0.279	-	-	-	0.224	-	-	-	0.271	-	-	-	-
**#6.**	-	0.392	-	-	-	0.527	-	-	-	0.257	-	-	-	0.397	-	-
**#7.**	-	0.56	-	-	-	0.542	-	-	-	0.564	-	-	-	0.58	-	-
**#8.**	-	0.529	-	-	-	0.487	-	-	-	0.437	-	-	-	0.564	-	-
**#9.**	0.295	-	-	-	-	-	-	0.239	-	-	0.2	-	0.43	-	-	-
**#10.**	0.799	-	-	-	0.809	-	-	-	0.774	-	-	-	0.799	-	-	-
**#11.**	0.815	-	-	-	0.784	-	-	-	0.792	-	-	-	0.836	-	-	-
	**Whole sample**				**Dijon**				**French Guiana**				**French West Indies**			
	**SW**	**LA**	**HS**	**FD**	**SW**	**LA**	**HS**	**FD**	**SW**	**LA**	**Dim 3**	**Dim 4**	**SW**	**LA**	**Dim 3**	**Dim 4**
**% of variance**	13	15.5	13.4	12.7	12	16.2	15.7	12.2	11.8	14.7	13.9	14.2	14.3	16	12.6	11.8
**#1.**	-	-	0.598	-	-	-	0.486	-	-	-	-	0.653	-	-	-	0.669
**#2.**	-	-	-	0.667	-	-	-	0.582	-	-	0.473	-	-	-	0.581	-
**#3.**	-	-	0.207	-	-	-	0.53	-	-	-	0.343	-	-	-	0.393	-
**#4.**	-	-	0.383	-	-	-	0.442	-	-	-	0.316	-	-	-	-	0.263
**#5.**	-	-	-	0.285	-	-	-	0.347	-	-	-	-	-	-	-	-
**#6.**	-	0.387	-	-	-	0.5	-	-	0.338	-	-	-	-	0.383	-	-
**#7.**	-	0.571	-	-	-	0.562	-	-	-	0.577	-	-	-	0.584	-	-
**#8.**	-	0.533	-	-	-	0.498	-	-	-	0.507	-	-	-	0.575	-	-
**#9.**	0.63	-	-	-	0.607	-	-	-	0.486	-	-	-	0.636	-	-	-
**#10.**	0.627	-	-	-	0.485	-	-	-	0.229	-	-	0.316	0.682	-	-	-

*SW*: *Social Withdrawal; LA*: *Leisures Activities; HS*: *Houshold Stability; FD*: *Financial difficulties; Dim*: *dimension (used when the dimension is not clearly identifiable);*

*For clarity purposes*, *only contributions higher than 0*.*2 are shown*

The two other dimensions were less consistent across groups, but were shared between the whole sample and the Dijon group: one comprised items #1, #3 and #4, and is thus related to household stability (HS), the others (items #2 and #5) are related to financial difficulties (FD). In the FWI group, these two dimensions were made up of items #1-#4, and #2-#3. In the FG group, there were also inconsistencies between the analysis of items #10 and #11. All these variations were not named and are referred to as dimensions 3 and 4 in Table 4.

### Impact of the DIF on the EPICES score and the latent variable estimation

**Fig 3:**
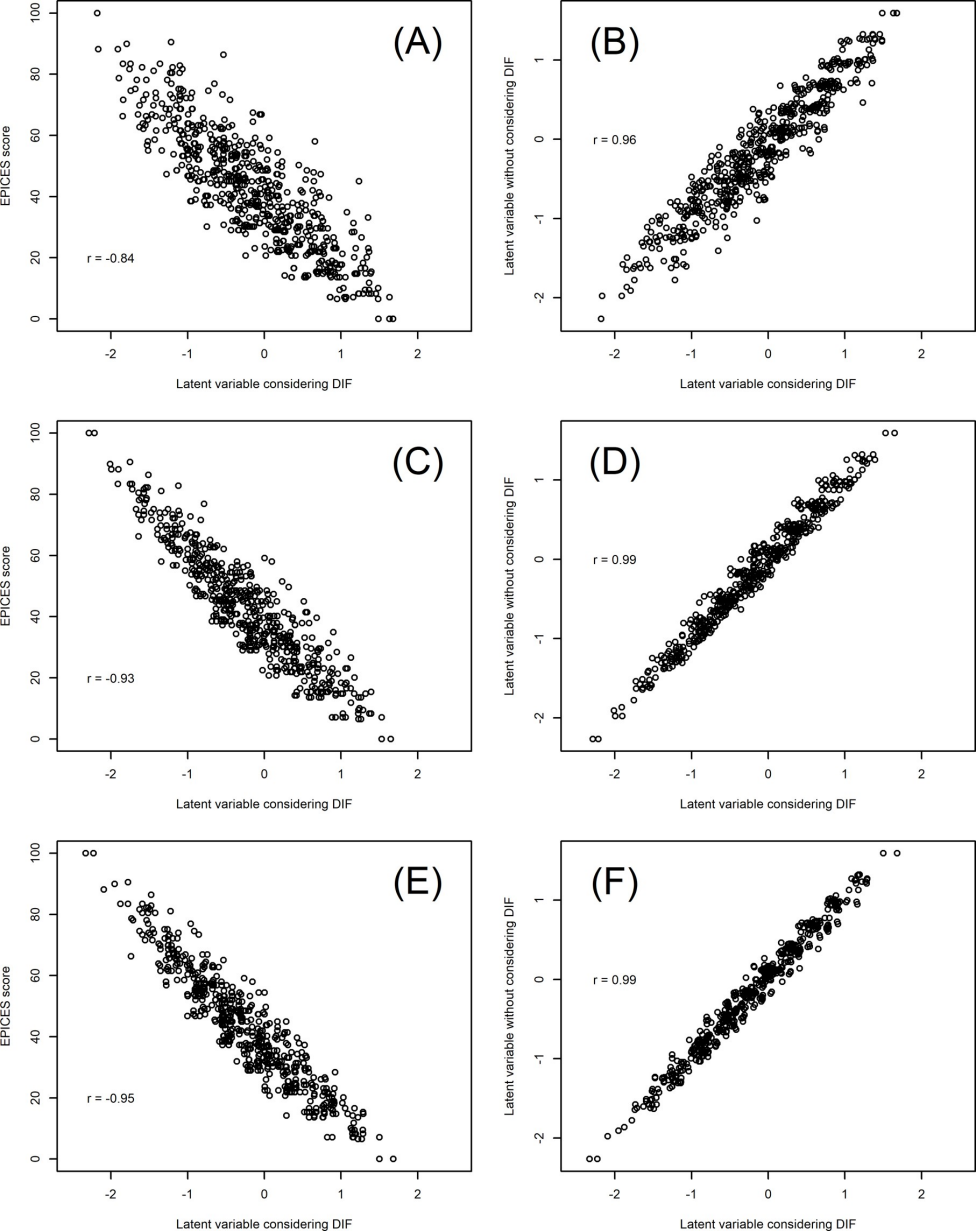
Correlation between EPICES score and estimated latent variable with and without DIFs.

Pearson correlations between the EPICES score and the latent variable resulting in the IRT analysis were high, even when considering DIF (Fig 3) between geographical areas (-0.84), gender (-0.93) and age categories (-0.95). The negative correlations are the result of scale differences between the two measurements, for the EPICES, a high score means a high level of precariousness, whereas for the individual latent measurement by the IRT, a high score means a low level of precariousness.

We also observed a strong correlation between the estimate of the latent factor with and without considering DIF (Fig 3) between geographical areas (0.96), gender (0.99) and age categories (0.99).

Using DIF due to geographical areas: fig A, B; Using DIF due to sex: Fig C, D; Using DIF due to age categories: Fig E, F

Pearson correlation between variable are directly rapported in fig

## Discussion

This work constitutes the first attempt to assess the DIF of the EPICES score between different French populations. Moderate or large DIF were observed between the FWI, FG and Dijon areas concerning five items: social workers, complementary health insurance, home-ownership, financial difficulties and sports. These differences could lead to the erroneous classification of patients, and need to be considered when using EPICES in heterogeneous population (as was the case in the INDIA study).

The main limit of our analysis is the study sample, which is not representative of the whole population of selected areas due to selection on stroke. As stroke happens relatively late in life, young people were not included in this analysis, thus DIF might be more present between young adults and older people than observed in our sample. Selection on stroke could also have led to a sample with higher cardiovascular risk factors, such as low physical activity. However, IRT analyses assess item’s difficulty independently from the sample characteristics, which should have limited the impact of having a highly-selected population.

Another limit is that we had to exclude item #11 due to a violation of local independence. The high residual correlations between the last two items are not surprising due to the similar wording. Either patients did not grasp the difference between “material assistance” and “shelter”, or the two items correspond to the same information. In our sample, about 93% of the patients answered the same to both items. Thus, it is likely that what was observed for item #10 could also have been observed for item #11. This limit should not have affected the DIF analysis, but it implies that our results are not directly applicable to the EPICES score. To measure precariousness or social deprivation in France it might be of interest to evaluate the psychometric characteristics of a simplified version of the EPICES score, or to develop a new score.

Explaining observed DIFs is a difficult task as it can be due to a difference in interpretation and/or in the socioeconomic and cultural context. In the INDIA study, the three areas studied differed significantly for socioeconomic context. In 2006, households with the same characteristics (age, household size, activities, education level) had a lower disposable income in FG (14.4% less) and the West Indies (Guadeloupe, 20.5% less; Martinique, 17.3% less) than in metropolitan France outside Paris [26]. Furthermore, commodities were around 12% more expensive in FG and FWI, in 2016, compared with metropolitan France [27], and unemployment was higher (in 2013: Guadeloupe, 26.1%; Martinique; 22.8%, FG, 21.3%, mainland France, 10%) [28]. In summary, populations in these overseas departments were significantly less favoured than those in mainland France.

Thus it was not surprising to observe five items with moderate to large DIFs by comparing regions (items #1, #2, #4, #5, #6). In the FWI, two items had a DIF with both mainland France and FG (“ever meet a social worker” and “home-ownership”), thus they were probably due to the local context. These items were almost not discriminant at all for the FWI, whereas this was not case for the other two regions. We also found these items in the last dimension of the MCA analysis for the FWI, while they were in separate dimensions for the other two regions. As such, it seems that this dimension in FWI represented something other than precariousness in the FWI.

Social worker in FWI reported a strong cultural taboo towards meeting or reporting a meeting with a social worker, which may explain the low discriminant capacity. Interpretation may also have been a cause of DIF, as the definition of ‘social worker’ could vary from one area to another.

For home-ownership, multiple reasons can explain the DIF due to the FWI context: high turnover of mainlanders, mostly civil servants coming from short term position with high salaries [29]. However, they rarely stay long in the FWI due to cultural differences and mainland roots. Thus, a non-negligible part of the population does not wish to acquire property even though they have the means to do so. Secondly, there was a higher percentage of people who owned their homes though inheritance in the FWI than in the mainland; the income for this part of the population was close to that of tenants of the private sector [30], significantly lower than in mainland. Jointly-owned land is commonplace due to family inheritances, and it may have increased the proportion of people in precarious situations even though they reported being a home owner. Finally, due to the historic context, there is a strong feeling of property even if the occupiers do not hold the deed, which may have led to an over-declaration of ownership [31].

In FG, two items had a DIF due to the local context: home-ownership and financial difficulties. Unlike DIF observed in FWI, they were uniform. Both DIFs can be explained jointly. As observed in the MCA analysis, EPICES is mostly a social deprivation index. Thus, the latent variable is not largely influenced by financial resources. As a result, and due to the higher cost of essentials in FG [27], it is not surprising to observe a higher probability of financial difficulties at the same level of the latent variable in FG compared to the others two areas. As property ownership is partly a financial matter (at least for those who have not inherited property), we can relate the higher level of financial difficulties to a lower level of home-ownership (at the same level of social deprivation).

Lastly, two DIF were due to the local context in mainland France compared with the other two areas: complementary health insurance and sports activities. DIF for complementary health insurance can be explained in the same way as that for home-ownership in FG: it is mostly a financial matter, as shown by the MCA analysis (financial difficulties and complementary health insurance shared the same dimension). Furthermore, a non-negligible part of the population in FG is from illegal immigration, living in poverty and without basic health insurance [32], they bring down the probability of having a complementary insurance for people in precariousness, compared to mainland. For sport activities, the difficulty was inversed: higher in the mainland than in the Caribbean region. Like for the social worker item, there is little in the literature to explain the difference. However, we observed a lower proportion of physical activity in Dijon (20%) than in FG (30%) or in the FWI (36%).

For comparisons between age categories and gender, there were significantly fewer items with at least moderate DIFs: one for age (item #3), two for gender (item #4 and #7), compensating for each other. For all comparisons (area, age categories, gender), seven of the ten items presented a differential item functioning, almost exclusively in the financial difficulties and household stability dimension. This indicates that these two dimensions are unstable and should be examined with caution in measuring precariousness or social deprivation across population with different socio-economic and cultural context. On the other hand, we did not observe much variation in the social withdrawal and leisure activities dimensions.

DIFs did not seem to have a strong impact on the EPICES score (as shown in Fig 3), which may be due to compensation between DIF. Furthermore, EPICES is often dichotomized using thresholds from the validation study. This limits classification bias, and thus the impact of observed DIFs in the main analysis of the INDIA study.

The low Cronbach alpha was also observed in the EPICES validation study [18]and was coherent with the MCA analysis. However, we did not observe any omitted dimensions in the PCA on residuals. As MCA and PCA analysis did not have the same goals (verify unidimensionality compared with analysing sub-dimensions), their results were not seen as contradictory.

The concept on which EPICES was designed, precariousness, anchored in the French context, remain a problem for its international use [33,34]. Still, as most items have also been used in other deprivation scores, with the exception of the last two, it could be viewed as a social deprivation score. This conclusion is reinforced by the MCA analysis, where we observed four subdimensions, of which three were close to the concept of social deprivation (social withdrawal, leisure activities and household stability), whereas the last one was closer to the notion of material deprivation or financial poverty (financial difficulties). EPICES also presents some limits in its construction (items redundancy and low internal consistency). However EPICES also have strong points: it is a short questionnaire, designed to be filled in a clinical context with limited time, which can also be useful in a research context. It was used successfully to analyse relationship between low socioeconomic status and health [14–17,35]. As such it seems worthy to be included in comparisons with other scales [36–38], before choosing a tool to measure precariousness or social deprivation at the individual level. As stated above, searchers in the field might consider improving the original score by validating a simplified version.

In conclusion, we found several items with differential functioning in the EPICES score. These DIFs were essentially present in the comparison of areas. This is not surprising given the socioeconomic and cultural differences between the areas studied. By analysing what could have caused these DIFs, it was evident that the wording of some items needs to be clarified (social worker and maybe sport activities), whereas for other items the DIF seems to be mainly the result of the local context (home-ownership and financial difficulties), which cannot be modified. However, the DIFs did not lead to a large difference between the latent variable and the EPICES score, which indicates that it can be used to assess precariousness and social deprivation between different areas of France and countries with similar socio-economic and cultural environment before the development of more validated score. More studies are needed to further ensure its stability, particularly in international comparisons.

## Supporting information

S1 Data (DOCX)
