# Supplementary material for: Social deprivation among socio-economic contrasted french areas: Using item response theory analysis to assess differential item functioning of the EPICES questionnaire in stroke patients
Source: PLoS One. 2020 Apr 2;15(4):e0230661. doi: 10.1371/journal.pone.0230661 (PMC7117693; doi:10.1371/journal.pone.0230661)
Supplement: S1 Data — (DOCX) [file pone.0230661.s001.docx]

**Supplementary material**

| **Dijon** | | | | | | | | | | | |
| --- | --- | --- | --- | --- | --- | --- | --- | --- | --- | --- | --- |
|  | 1 | 2 | 3 | 4 | 5 | 6 | 7 | 8 | 9 | 10 | 11 |
| 1 | - | **0.418** | 0.268 | 0.159 | 0.257 | 0.146 | -0.149 | -0.101 | 0.295 | 0.093 | -0.021 |
| 2 |  | - | 0.161 | 0.110 | **0.381** | 0.263 | -0.102 | -0.105 | **0.423** | 0.282 | 0.159 |
| 3 |  |  | - | 0.174 | 0.047 | 0.015 | -0.138 | -0.083 | 0.110 | -0.085 | -0.097 |
| 4 |  |  |  | - | 0.179 | -0.094 | -0.241 | -0.166 | 0.022 | -0.053 | -0.093 |
| 5 |  |  |  |  | - | 0.076 | -0.162 | -0.196 | 0.218 | 0.170 | 0.076 |
| 6 |  |  |  |  |  | - | 0.053 | 0.035 | 0.189 | -0.069 | -0.188 |
| 7 |  |  |  |  |  |  | - | 0.025 | -0.137 | **-0.308** | **-0.347** |
| 8 |  |  |  |  |  |  |  | - | -0.084 | -0.296 | **-0.361** |
| 9 |  |  |  |  |  |  |  |  | - | 0.251 | 0.109 |
| 10 |  |  |  |  |  |  |  |  |  | - | **0.558** |
| 11 |  |  |  |  |  |  |  |  |  |  | - |
| **French guyana** | | | | | | | | | | | |
|  | 1 | 2 | 3 | 4 | 5 | 6 | 7 | 8 | 9 | 10 | 11 |
| 1 | - | 0.017 | 0.125 | 0.136 | 0.092 | 0.279 | 0.025 | -0.049 | 0.254 | 0.106 | -0.011 |
| 2 |  | - | 0.069 | 0.108 | 0.126 | 0.186 | -0.019 | 0.033 | 0.135 | -0.140 | -0.181 |
| 3 |  |  | - | 0.145 | 0.009 | 0.137 | -0.044 | 0.039 | 0.123 | -0.189 | -0.175 |
| 4 |  |  |  | - | 0.002 | 0.053 | -0.164 | -0.149 | 0.061 | -0.098 | -0.117 |
| 5 |  |  |  |  | - | 0.080 | -0.054 | -0.113 | -0.056 | -0.214 | -0.233 |
| 6 |  |  |  |  |  | - | 0.287 | 0.118 | 0.187 | -0.048 | -0.131 |
| 7 |  |  |  |  |  |  | - | 0.183 | -0.028 | -0.260 | -0.293 |
| 8 |  |  |  |  |  |  |  | - | 0.015 | -0.287 | -0.293 |
| 9 |  |  |  |  |  |  |  |  | - | 0.072 | 0.077 |
| 10 |  |  |  |  |  |  |  |  |  | - | **0.525** |
| 11 |  |  |  |  |  |  |  |  |  |  | - |
| **West indies** | | | | | | | | | | | |
|  | 1 | 2 | 3 | 4 | 5 | 6 | 7 | 8 | 9 | 10 | 11 |
| 1 | - | **0.450** | 0.240 | **0.406** | **0.319** | -0.016 | 0.073 | 0.115 | 0.258 | 0.092 | 0.104 |
| 2 |  | - | 0.239 | 0.274 | 0.241 | -0.051 | 0.048 | 0.090 | 0.149 | 0.017 | 0.043 |
| 3 |  |  | - | 0.216 | 0.147 | -0.056 | -0.103 | -0.032 | -0.008 | -0.083 | -0.125 |
| 4 |  |  |  | - | 0.229 | -0.096 | -0.123 | -0.003 | 0.092 | 0.083 | 0.092 |
| 5 |  |  |  |  | - | -0.167 | -0.092 | -0.096 | 0.007 | 0.020 | 0.019 |
| 6 |  |  |  |  |  | - | -0.057 | -0.030 | -0.265 | **-0.335** | **-0.350** |
| 7 |  |  |  |  |  |  | - | 0.211 | -0.188 | **-0.309** | -0.293 |
| 8 |  |  |  |  |  |  |  | - | -0.188 | -0.291 | -0.294 |
| 9 |  |  |  |  |  |  |  |  | - | 0.171 | 0.254 |
| 10 |  |  |  |  |  |  |  |  |  | - | **0.662** |
| 11 |  |  |  |  |  |  |  |  |  |  | - |

S1: Correlations between residuals to assess local independence by geographical areas

*Correlations with absolute value superior to 0.3 were shown in bold*

| **Women** | | | | | | | | | | | |
| --- | --- | --- | --- | --- | --- | --- | --- | --- | --- | --- | --- |
|  | 1 | 2 | 3 | 4 | 5 | 6 | 7 | 8 | 9 | 10 | 11 |
| 1 | - | **0.341** | 0.154 | 0.229 | 0.232 | 0.086 | -0.111 | -0.013 | 0.246 | 0.075 | 0.079 |
| 2 |  | - | 0.209 | 0.167 | 0.231 | 0.147 | -0.048 | 0.019 | 0.280 | 0.091 | 0.109 |
| 3 |  |  | - | 0.090 | 0.068 | 0.005 | -0.071 | -0.029 | 0.078 | -0.056 | -0.124 |
| 4 |  |  |  | - | 0.154 | -0.081 | -0.203 | -0.110 | 0.059 | 0.008 | 0.004 |
| 5 |  |  |  |  | - | -0.033 | -0.127 | -0.150 | 0.032 | 0.044 | 0.028 |
| 6 |  |  |  |  |  | - | 0.033 | 0.057 | 0.016 | -0.173 | -0.173 |
| 7 |  |  |  |  |  |  | - | 0.156 | -0.157 | **-0.353** | **-0.329** |
| 8 |  |  |  |  |  |  |  | - | -0.051 | -0.267 | -0.258 |
| 9 |  |  |  |  |  |  |  |  | - | 0.204 | 0.210 |
| 10 |  |  |  |  |  |  |  |  |  | - | **0.516** |
| 11 |  |  |  |  |  |  |  |  |  |  | - |
| **Men** | | | | | | | | | | | |
|  | 1 | 2 | 3 | 4 | 5 | 6 | 7 | 8 | 9 | 10 | 11 |
| 1 | - | 0.265 | 0.173 | 0.246 | 0.179 | 0.206 | 0.040 | -0.010 | 0.275 | 0.097 | 0.013 |
| 2 |  | - | 0.199 | 0.141 | 0.211 | 0.011 | 0.000 | -0.018 | 0.082 | -0.044 | -0.124 |
| 3 |  |  | - | 0.196 | 0.067 | -0.026 | -0.121 | -0.098 | 0.008 | -0.192 | -0.163 |
| 4 |  |  |  | - | 0.150 | -0.006 | -0.194 | -0.144 | -0.030 | -0.071 | -0.083 |
| 5 |  |  |  |  | - | -0.064 | -0.110 | -0.166 | -0.045 | -0.085 | -0.132 |
| 6 |  |  |  |  |  | - | 0.128 | 0.066 | 0.044 | -0.118 | -0.190 |
| 7 |  |  |  |  |  |  | - | 0.174 | -0.133 | -0.238 | -0.291 |
| 8 |  |  |  |  |  |  |  | - | -0.187 | **-0.320** | **-0.378** |
| 9 |  |  |  |  |  |  |  |  | - | 0.152 | 0.137 |
| 10 |  |  |  |  |  |  |  |  |  | - | **0.669** |
| 11 |  |  |  |  |  |  |  |  |  |  | - |

S2: Correlations between residuals to assess local independence by gender

*Correlations with absolute value superior to 0.3 were shown in bold*

| **< 65 years old** | | | | | | | | | | | |
| --- | --- | --- | --- | --- | --- | --- | --- | --- | --- | --- | --- |
|  | 1 | 2 | 3 | 4 | 5 | 6 | 7 | 8 | 9 | 10 | 11 |
| 1 | - | 0.264 | 0.196 | 0.173 | 0.140 | 0.078 | -0.053 | -0.136 | 0.287 | 0.109 | 0.023 |
| 2 |  | - | 0.202 | 0.093 | 0.108 | -0.010 | -0.067 | -0.110 | 0.198 | -0.006 | -0.076 |
| 3 |  |  | - | 0.126 | 0.048 | -0.024 | -0.071 | -0.108 | 0.055 | -0.132 | -0.159 |
| 4 |  |  |  | - | 0.007 | -0.018 | -0.173 | -0.102 | 0.024 | -0.079 | -0.111 |
| 5 |  |  |  |  | - | -0.034 | -0.082 | -0.227 | -0.048 | -0.119 | -0.198 |
| 6 |  |  |  |  |  | - | -0.043 | -0.131 | 0.028 | -0.135 | -0.183 |
| 7 |  |  |  |  |  |  | - | 0.059 | -0.155 | -0.258 | -0.270 |
| 8 |  |  |  |  |  |  |  | - | -0.189 | **-0.340** | **-0.341** |
| 9 |  |  |  |  |  |  |  |  | - | 0.207 | 0.199 |
| 10 |  |  |  |  |  |  |  |  |  | - | **0.514** |
| 11 |  |  |  |  |  |  |  |  |  |  | - |
| **≥ 65 years old** | | | | | | | | | | | |
|  | 1 | 2 | 3 | 4 | 5 | 6 | 7 | 8 | 9 | 10 | 11 |
| 1 | - | **0.317** | 0.135 | 0.262 | 0.216 | 0.276 | 0.080 | 0.162 | 0.233 | 0.068 | 0.052 |
| 2 |  | - | 0.125 | 0.157 | 0.274 | 0.196 | 0.122 | 0.165 | 0.145 | 0.033 | 0.024 |
| 3 |  |  | - | 0.132 | 0.019 | 0.027 | -0.095 | -0.001 | -0.034 | -0.154 | -0.185 |
| 4 |  |  |  | - | 0.119 | 0.032 | -0.113 | -0.040 | -0.056 | -0.070 | -0.071 |
| 5 |  |  |  |  | - | 0.013 | -0.035 | -0.021 | -0.033 | -0.021 | -0.021 |
| 6 |  |  |  |  |  | - | 0.209 | 0.218 | 0.063 | -0.090 | -0.117 |
| 7 |  |  |  |  |  |  | - | 0.262 | -0.080 | -0.228 | -0.237 |
| 8 |  |  |  |  |  |  |  | - | -0.028 | -0.183 | -0.212 |
| 9 |  |  |  |  |  |  |  |  | - | 0.139 | 0.143 |
| 10 |  |  |  |  |  |  |  |  |  | - | **0.670** |
| 11 |  |  |  |  |  |  |  |  |  |  | - |

S3: Correlations between residuals to assess local independence by age categories

*Correlations with absolute value superior to 0.3 were shown in bold*

|  | 1 | 2 | 3 | 4 | 5 | 6 | 7 | 8 | 9 | 10 |
| --- | --- | --- | --- | --- | --- | --- | --- | --- | --- | --- |
| 1 | - | 0.210 | 0.068 | 0.228 | 0.157 | 0.056 | -0.174 | -0.208 | 0.280 | 0.237 |
| 2 |  | - | 0.061 | 0.143 | 0.189 | -0.049 | -0.148 | -0.180 | 0.167 | 0.132 |
| 3 |  |  | - | 0.105 | -0.017 | -0.114 | -0.253 | -0.241 | -0.023 | -0.076 |
| 4 |  |  |  | - | 0.164 | -0.088 | -0.269 | -0.231 | 0.058 | 0.116 |
| 5 |  |  |  |  | - | -0.120 | -0.216 | **-0.300** | 0.017 | 0.102 |
| 6 |  |  |  |  |  | - | -0.031 | -0.103 | -0.007 | -0.064 |
| 7 |  |  |  |  |  |  | - | -0.023 | -0.203 | -0.229 |
| 8 |  |  |  |  |  |  |  | - | -0.248 | **-0.306** |
| 9 |  |  |  |  |  |  |  |  | - | **0.342** |
| 10 |  |  |  |  |  |  |  |  |  | - |

S4: Correlations between residuals to assess local independence in the whole population after excluding item 11

*Correlations with absolute value superior to 0.3 were shown in bold*

| **Dijon** | | | | | | | | | | |
| --- | --- | --- | --- | --- | --- | --- | --- | --- | --- | --- |
|  | 1 | 2 | 3 | 4 | 5 | 6 | 7 | 8 | 9 | 10 |
| 1 | - | **0.324** | 0.092 | 0.073 | 0.185 | -0.085 | -0.275 | **-0.324** | 0.220 | 0.201 |
| 2 |  | - | 0.029 | 0.082 | **0.404** | 0.154 | -0.114 | -0.218 | **0.453** | **0.511** |
| 3 |  |  | - | 0.074 | -0.071 | -0.207 | -0.276 | **-0.300** | -0.008 | -0.043 |
| 4 |  |  |  | - | 0.166 | -0.226 | -0.280 | -0.291 | 0.009 | 0.090 |
| 5 |  |  |  |  | - | -0.030 | -0.190 | **-0.314** | 0.209 | **0.341** |
| 6 |  |  |  |  |  | - | -0.036 | -0.127 | 0.086 | 0.015 |
| 7 |  |  |  |  |  |  | - | -0.068 | -0.163 | -0.157 |
| 8 |  |  |  |  |  |  |  | - | -0.200 | -0.235 |
| 9 |  |  |  |  |  |  |  |  | - | **0.412** |
| 10 |  |  |  |  |  |  |  |  |  | - |
| **French Guyana** | | | | | | | | | | |
|  | 1 | 2 | 3 | 4 | 5 | 6 | 7 | 8 | 9 | 10 |
| 1 | - | -0.139 | -0.000 | -0.010 | -0.126 | 0.122 | -0.100 | -0.194 | 0.181 | 0.241 |
| 2 |  | - | -0.011 | 0.013 | -0.026 | 0.065 | -0.122 | -0.071 | 0.065 | -0.074 |
| 3 |  |  | - | 0.066 | -0.145 | 0.017 | -0.149 | -0.078 | 0.062 | -0.118 |
| 4 |  |  |  | - | -0.137 | -0.062 | -0.250 | -0.243 | -0.015 | -0.009 |
| 5 |  |  |  |  | - | -0.063 | -0.197 | -0.262 | -0.195 | -0.187 |
| 6 |  |  |  |  |  | - | 0.204 | -0.002 | 0.107 | 0.017 |
| 7 |  |  |  |  |  |  | - | 0.110 | -0.051 | -0.086 |
| 8 |  |  |  |  |  |  |  | - | -0.024 | -0.156 |
| 9 |  |  |  |  |  |  |  |  | - | 0.276 |
| 10 |  |  |  |  |  |  |  |  |  | - |
| **West indies** | | | | | | | | | | |
|  | 1 | 2 | 3 | 4 | 5 | 6 | 7 | 8 | 9 | 10 |
| 1 | - | **-0.413** | -0.174 | **-0.388** | **-0.317** | 0.062 | 0.054 | 0.092 | **-0.327** | -0.294 |
| 2 |  | - | 0.189 | 0.298 | 0.276 | -0.108 | -0.140 | -0.073 | 0.178 | 0.149 |
| 3 |  |  | - | 0.242 | 0.172 | -0.082 | -0.242 | -0.159 | 0.051 | 0.036 |
| 4 |  |  |  | - | **0.331** | -0.069 | -0.199 | -0.060 | 0.221 | 0.266 |
| 5 |  |  |  |  | - | -0.106 | -0.148 | -0.136 | 0.171 | 0.224 |
| 6 |  |  |  |  |  | - | -0.212 | -0.179 | -0.222 | -0.202 |
| 7 |  |  |  |  |  |  | - | -0.064 | **-0.313** | **-0.344** |
| 8 |  |  |  |  |  |  |  | - | **-0.303** | **-0.318** |
| 9 |  |  |  |  |  |  |  |  | - | **0.361** |
| 10 |  |  |  |  |  |  |  |  |  | - |

S5: Correlations between residuals to assess local independence by geographical areas after excluding item 11

*Correlations with absolute value superior to 0.3 were shown in bold*

| **Women** | | | | | | | | | | |
| --- | --- | --- | --- | --- | --- | --- | --- | --- | --- | --- |
|  | 1 | 2 | 3 | 4 | 5 | 6 | 7 | 8 | 9 | 10 |
| 1 | - | 0.296 | 0.071 | 0.217 | 0.239 | 0.050 | -0.284 | -0.197 | 0.233 | 0.209 |
| 2 |  | - | 0.140 | 0.162 | 0.246 | 0.128 | -0.186 | -0.135 | 0.277 | 0.231 |
| 3 |  |  | - | 0.047 | 0.040 | -0.071 | -0.227 | -0.199 | 0.016 | 0.008 |
| 4 |  |  |  | - | 0.186 | -0.086 | **-0.309** | -0.216 | 0.073 | 0.134 |
| 5 |  |  |  |  | - | -0.020 | -0.201 | -0.243 | 0.070 | 0.191 |
| 6 |  |  |  |  |  | - | -0.094 | -0.066 | 0.012 | -0.022 |
| 7 |  |  |  |  |  |  | - | -0.055 | **-0.300** | **-0.340** |
| 8 |  |  |  |  |  |  |  | - | -0.214 | -0.282 |
| 9 |  |  |  |  |  |  |  |  | - | **0.355** |
| 10 |  |  |  |  |  |  |  |  |  | - |
| **Men** | | | | | | | | | | |
|  | 1 | 2 | 3 | 4 | 5 | 6 | 7 | 8 | 9 | 10 |
| 1 | - | 0.087 | -0.006 | 0.162 | 0.056 | 0.158 | -0.022 | -0.146 | **0.350** | **0.306** |
| 2 |  | - | 0.002 | -0.003 | 0.060 | -0.132 | -0.154 | -0.228 | 0.031 | 0.007 |
| 3 |  |  | - | 0.066 | -0.127 | -0.160 | -0.291 | **-0.323** | -0.051 | -0.160 |
| 4 |  |  |  | - | 0.051 | -0.074 | -0.285 | -0.278 | -0.015 | 0.033 |
| 5 |  |  |  |  | - | -0.153 | -0.228 | **-0.334** | -0.055 | 0.005 |
| 6 |  |  |  |  |  | - | 0.109 | 0.009 | 0.098 | 0.040 |
| 7 |  |  |  |  |  |  | - | 0.085 | -0.071 | -0.068 |
| 8 |  |  |  |  |  |  |  | - | -0.192 | -0.219 |
| 9 |  |  |  |  |  |  |  |  | - | **0.389** |
| 10 |  |  |  |  |  |  |  |  |  | - |

S6: Correlations between residuals to assess local independence by gender after excluding item 11

*Correlations with absolute value superior to 0.3 were shown in bold*

| **< 65 years old** | | | | | | | | | | |
| --- | --- | --- | --- | --- | --- | --- | --- | --- | --- | --- |
|  | 1 | 2 | 3 | 4 | 5 | 6 | 7 | 8 | 9 | 10 |
| 1 | - | 0.155 | 0.079 | 0.112 | 0.049 | 0.070 | -0.196 | -0.270 | 0.273 | 0.213 |
| 2 |  | - | 0.092 | 0.021 | 0.016 | -0.051 | -0.206 | -0.246 | 0.143 | 0.044 |
| 3 |  |  | - | 0.040 | -0.064 | -0.072 | -0.215 | -0.262 | -0.019 | -0.115 |
| 4 |  |  |  | - | -0.058 | -0.029 | -0.275 | -0.196 | 0.002 | -0.007 |
| 5 |  |  |  |  | - | -0.052 | -0.208 | **-0.357** | -0.091 | -0.073 |
| 6 |  |  |  |  |  | - | -0.085 | -0.161 | 0.071 | 0.011 |
| 7 |  |  |  |  |  |  | - | -0.055 | -0.236 | -0.224 |
| 8 |  |  |  |  |  |  |  | - | -0.257 | -0.296 |
| 9 |  |  |  |  |  |  |  |  | - | **0.352** |
| 10 |  |  |  |  |  |  |  |  |  | - |
| **≥ 65 years old** | | | | | | | | | | |
|  | 1 | 2 | 3 | 4 | 5 | 6 | 7 | 8 | 9 | 10 |
| 1 | - | 0.189 | -0.006 | 0.124 | 0.130 | 0.097 | -0.033 | -0.070 | **0.308** | 0.252 |
| 2 |  | - | 0.006 | 0.023 | 0.221 | 0.029 | 0.022 | -0.037 | 0.207 | 0.190 |
| 3 |  |  | - | 0.011 | -0.077 | -0.138 | -0.234 | -0.204 | -0.025 | -0.068 |
| 4 |  |  |  | - | 0.021 | -0.177 | -0.271 | -0.288 | -0.037 | 0.022 |
| 5 |  |  |  |  | - | -0.122 | -0.109 | -0.192 | 0.043 | 0.146 |
| 6 |  |  |  |  |  | - | 0.101 | 0.037 | 0.052 | -0.001 |
| 7 |  |  |  |  |  |  | - | 0.104 | -0.007 | -0.055 |
| 8 |  |  |  |  |  |  |  | - | -0.084 | -0.153 |
| 9 |  |  |  |  |  |  |  |  | - | **0.398** |
| 10 |  |  |  |  |  |  |  |  |  | - |

S6: Correlations between residuals to assess local independence by age categories after excluding item 11

*Correlations with absolute value superior to 0.3 were shown in bold*
